# Quantity and location of aortic valve calcification predicts paravalvular leakage after transcatheter aortic valve replacement: a systematic review and meta-analysis

**DOI:** 10.3389/fcvm.2023.1170979

**Published:** 2023-05-24

**Authors:** Jiale Shi, Wei Li, Tangshan Zhang, Chengwen Han, Zhengjun Wang, Xinhao Pei, Xuetao Li, Zidong Zhao, Pengbo Wang, Jingying Han, Shiqiao Chen

**Affiliations:** ^1^ Shandong Provincial Hospital, Shandong University, Jinan, China; ^2^ Department of Second Clinical Medical School, Cheeloo College of Medicine, Shandong University, Jinan, China; ^3^ Department of Basic Medical School, Cheeloo College of Medicine, Shandong University, Jinan, China; ^4^ Department of Vascular Surgery, Jiyang District People's Hospital, Jinan, China; ^5^ Department of Cardiovascular Surgery, Shandong Provincial Hospital Affiliated to Shandong First Medical University, Jinan, China; ^6^ Department of Public Health School, Cheeloo College of Medicine, Shandong University, Jinan, China; ^7^ Department of Interventional Diagnosis and Treatment, Shandong Provincial Hospital Affiliated to Shandong First Medical University, Jinan, China

**Keywords:** paravalvular leakage (PVL), transcatheter aortic valve replacement (TAVI), aortic valve complex, calcification, left ventricular outflow tract (LVOT)

## Abstract

**Introduction:**

Transcatheter aortic valve replacement (TAVR) is the first-line treatment for patients with moderate-to-high surgical risk of severe aortic stenosis. Paravalvular leakage (PVL) is a serious complication of TAVR, and aortic valve calcification contributes to the occurrence of PVL. This study aimed to investigate the effect of location and quantity of calcification in the aortic valve complex (AVC) and left ventricular outflow tract (LVOT) on PVL after TAVR.

**Method:**

We performed a systematic review and meta-analysis to evaluate the effect of quantity and location of aortic valve calcification on PVL after TAVR using observational studies from PubMed and EMBASE databases from inception to February 16, 2022.

**Results:**

Twenty-four observational studies with 6,846 patients were included in the analysis. A high quantity of calcium was observed in 29.6% of the patients; they showed a higher risk of significant PVL. There was heterogeneity between studies (I2 = 15%). In the subgroup analysis, PVL after TAVR was associated with the quantity of aortic valve calcification, especially those located in the LVOT, valve leaflets, and the device landing zone. A high quantity of calcium was associated with PVL, regardless of expandable types or MDCT thresholds used. However, for valves with sealing skirt, the amount of calcium has no significant effect on the incidence of PVL.

**Conclusion:**

Our study elucidated the effect of aortic valve calcification on PVL and showed that the quantity and location of aortic valve calcification can help predict PVL. Furthermore, our results provide a reference for the selection of MDCT thresholds before TAVR. We also showed that balloon-expandable valves may not be effective in patients with high calcification, and valves with sealing skirts instead of those without sealing skirts should be applied more to prevent PVL from happening.

**Systematic Review Registration:**

https://www.crd.york.ac.uk/PROSPERO/display_record.php?RecordID=354630, identifier: CRD42022354630.

## 1. Introduction

Transcatheter aortic valve replacement (TAVR) is the first-line treatment for patients with moderate-to-high surgical risk of severe aortic stenosis (1–3). Paravalvular leakage (PVL) is a serious complication of TAVR, which leads to an increased mortality rate (4–6). Aortic valve calcification is a factor contributing to the occurrence of PVL (7, 8); however, the location and pattern of calcification vary greatly between patients. Furthermore, the effects of calcification in various locations of the aortic valve complex (AVC) and left ventricular outflow tract (LVOT) are not fully understood. We performed a systematic review and meta-analysis to investigate the effect of location and quantity of calcification in the AVC and LVOT on PVL after TAVR. The present study was registered with PROSPERO (CRD42022354630). This manuscript is written in accordance with the Preferred Reported Items for Systematic Reviews and Meta-Analysis (PRISMA) checklist.

## 2. Systematic review

A systematic review of published data on the quantity and location of aortic valve calcification in patients who had undergone TAVR, and on the incidence of PVL after TAVR was conducted using PubMed and EMBASE databases. The guidance and reporting items specified in the PRISMA statement were adhered to (9). The following MeSH terms were used: transcatheter aortic valve replacement and calcium. The following keywords were used: paravalvular leakage and paravalvular regurgitation.

The databases were last accessed on February 16, 2022. The citations were screened at the title and abstract level, and the full text was retrieved if the relationship between calcification and PVL at either region of the aortic valve was reported. Studies that met the following criteria were included: (i) original design; (ii) reported data on the occurrence of PVL in patients after TAVR; (iii) quantity and specific location of calcification in the aortic valve of selected patients. When two similar studies were reported by the same institution or author, the most recent publication or one with additional information was included in the analysis. Case reports or studies published in languages other than English were excluded.

A standardized data summary form was used to extract data from patients and studies. Two investigators (LW and PXH) performed the data extraction twice. When necessary, consensus was reached with the help of a third investigator (SJL) to resolve any disagreements. When the precise data for the study were not available, the corresponding author was contacted, and additional information was requested.

Using the above criteria, we extracted the following data: (1) general characteristics (i.e., name of the authors, year of study, region, inclusion period, and sample size), (2) TAVR procedure (i.e., valve and expandable type), (3) characteristics of the calcification [i.e., multidetector computed tomography (MDCT) examination threshold, area scanned, and calcium volume or score], and (4) characteristics of PVL (i.e., extent and time definition).

We analyzed the data using Review Manager version 5.3 (RevMan; Copenhagen: The Nordic Cochrane Centre, The Cochrane Collaboration, 2014) and estimated the relative risk (RR), odds ratios (OR), as well as the standardized mean differences (SMD), with 95% confidence intervals (CI) for all available categorical and continuous variables. Given the possible heterogeneity in outcome ascertainment across trials, we analyzed our data based on SMD values as they provided a more comprehensive summary statistic on the size of the intervention effect in each study relative to the variability observed in that study. Continuous variables, which were reported as medians with the first and third quartile of the sample, were converted to mean and standard deviation values according to the method described by Luo et al. (10) and Wan et al. (11). The *I*^2^ index was used to assess the consistency across studies. The choice between a random- or fixed-effect model was not made based on the degree of heterogeneity but according to the recent recommendations outlined by the American Heart Association (12) (i.e., by determining the functional similarity between the included studies and estimating a common effect size that would apply to populations similar to those included in the meta-analysis).

To assess the potential effect of publication bias, we examined the funnel plots for asymmetry (Supplementary Figures S1–4). The risk of bias was evaluated through Revman. In addition, sub-analyses were performed to assess the effect of location, expandable type, and MDCT-detected threshold on the relationship between the quantity of calcium and PVL.

**Figure 1:**
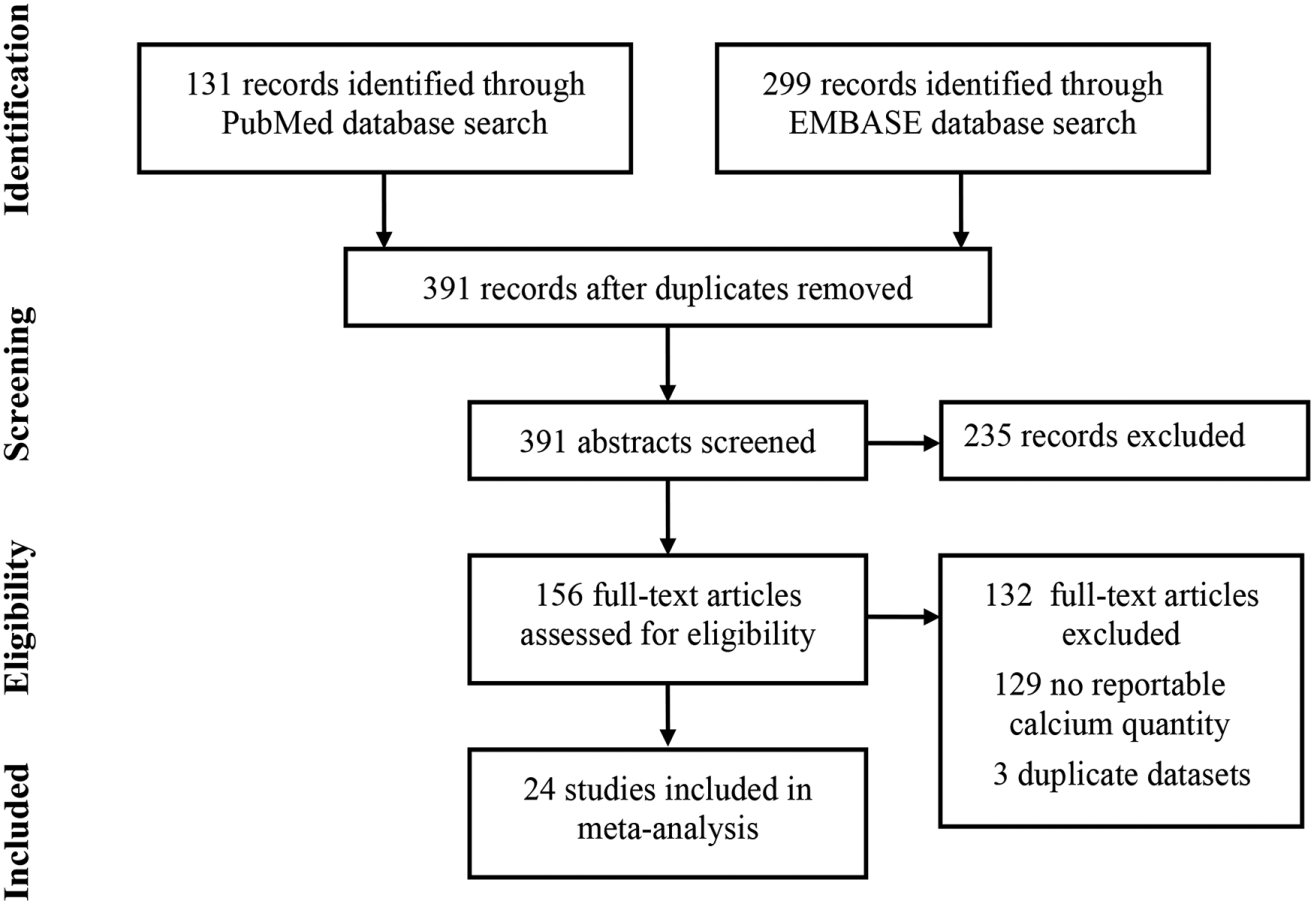
Flow chart of selected studies. Flow chart of studies evaluating the effect quantity and location of aortic valve calcification on paravalvular leakage (PVL) in transcatheter aortic valve replacement (TAVR) recipients. The studies were selected based on the Preferred Reported Items for Systematic Reviews and Meta-Analysis (PRISMA) statement.

The systematic search using PubMed and EMBASE databases yielded 131 and 299 records, respectively, with 391 total records, which were reviewed at the title and abstract level after excluding duplicates. Of those, 159 were selected and assessed for eligibility at the full-text level, 132 were excluded from the quantitative analysis owing to missing detailed data on calcium quantity, and three were excluded due to duplicated datasets. This review included 24 studies in the final analysis (Figure 1), with an overall sample of 6,846 patients (Table 1). The risk of bias (13) for each included study was evaluated with Revman, and no significant bias was found. The calcium quantity was expressed as calcium volume or score, which was detected by MDCT using patient-specific detection or fixed threshold. The regions of interest, including the aortic valve leaflets, annulus, and LVOT, were detected similarly using MDCT. Two regions were defined as follows: AVC, containing the annulus and leaflets, and the device landing zone (DLZ), containing the AVC and LVOT (14, 15). The PVL was assessed using echocardiography after the procedure, at discharge, or after a month. PVL was classified into four grades: absent (0), trace or mild (1/4), mild-to-moderate (2/4), moderate-to-severe (3/4), and severe (4/4). We regarded paravalvular leakage ≥2/4 as significant (16). New generation valves referred to prosthetic valves with sealing skirts (17–19), which could fit to the wall of the blood vessel, such as outer skirt or downward skirt, while old generations had no sealing skirts.

**Table 1:** Summary of studies evaluating the effect of aortic valve calcification on PVL after TAVR.

Study	Year	Region	Sample size	Age	Female	Inclusion period	Valve type	Expandable type	Implantation approach	PVL definition
Abramowitz et al.	2016	United States	299	83.2 ± 7.8	36.0%	April 2012 to April 2014	SAPIEN (45%) SAPIEN XT (57%)	Balloon-expandable (100%)	Transfemoral (77.6%) transapical (8.4%) transaortic (14%)	One month after operation
Bettinger et al.	2017	NA	104	83 ± 9	54.0%	May 2014 to July 2015	CoreValve (100%)	Self-expanding valve (100%)	Transfemoral (98.1%) transaortic (1.8%)	Post-procedural
Fonseca et al.	2016	Portugal	152	79.4 ± 6.7	53.9%	August 2007 to November 2014	CoreValve (67.8%) SAPIEN XT (21.2%) SAPIEN 3 (11%)	Self-expanding (67.8%) balloon-expandable (32.2%)	Transfemoral (84.2%) transsubclavian (7.9%) transapical (5.9%) transaortic (2%)	Post-procedural
Gorla et al.	2021	Italy	359	83.2 ± 6.4	50.4%	2016 to 2019	Evolut R (100%)	Self-expanding (100%)	Transfemoral (91.4%) transsubclavian (8.6%)	Post-procedural
Hagar et al.	2020	China	256	74 ± 6	43.4%	April 2012 to November 2017	CoreValve (NA) Venus A (NA) VitaFlow (NA) Taurus One (NA) Lotus Valve (12.5%) SAPIEN XT (NA) SAPIEN 3 (NA)	Self-expanding (83.2%) mechanically expandable (12.5%) balloon expandable (4.3%)	Transfemoral (99.2%) transsubclavian (0.4%) transcarotid (0.4%)	Post-procedural
Hansson et al.	2018	Denmark Canada and United Kingdom	302	82.9 ± 1.39	50.0%	August 2011 to February 2014	SAPIEN XT (100%)	Balloon-expandable (100%)	NA	Discharge
Jilaihawi et al.	2014	Europe	198	86.0 ± 1.65	50.0%	November 2007 to April 2012	SAPIEN (NA) SAPIEN XT (NA)	Balloon-expandable (100%)	Transfemoral (87.4%) transapical (12.6%)	Post-procedural
Jochheim et al.	2020	Germany	690	80.8 ± 7.2	50.4%	January 2013 to December 2015	SAPIEN S3 (66.0%) SAPIEN XT (19.1%) CoreValve E (7.2%) Lotus (7.7%)	Balloon-expandable (79.1%) self-expanding (7.2%) mechanically expandable (7.7%)	Transfemoral (100%)	One month after operation
Kaneko et al.	2017	Germany	281	80 ± 6	47.0%	February 2014 to December 2015	SAPIEN 3 (100%)	Balloon-expandable (100%)	Transfemoral (100%)	Discharge
Khalique et al.	2014	United States	150	83.3 ± 8.2	54.0%	October 2011 to July 2013	SAPIEN THV (NA) SAPIEN XT (NA)	Balloon-expandable (100%)	Transfemoral (83%) transaortic (9%) transapical (8%)	Post-procedural
Ki et al.	2020	South Korea	287	78.5 ± 1.6	47.5%	July 2011 to February 2020	SAPIEN (NA) SAPIEN XT (NA) SAPIEN 3 (NA) CoreValve (NA) Evolut R (NA) Evolut Pro (NA) Lotus (NA) Lotus Edge (NA)	Self-expanding (NA) balloon-expandable (NA) mechanically expandable (NA)	Transfemoral (98.3%) transapical (1.7%)	One month after operation
Kim et al.	2018	Europe	1,000	81.1 ± 5.2	61.2%	October 2014 to April 2016	Acurate neo (100%)	Self-expanding (100%)	Transfemoral (100%)	One year after operation
Kim et al.	2018	Germany	141	81.3 ± 5.7	56.0%	June to December 2016	SAPIEN 3 (48.9%) Acurate neo (51.1%)	Balloon-expandable (48.9%) self-expanding (51.1%)	Transfemoral (100%)	Post-procedural
Ko et al.	2021	South Korea	676	79.8 ± 5.4	49.3%	March 2010 to December 2019	SAPIEN (0.9%) SAPIEN XT (17.2%) SAPIEN 3 (58.7%) CoreValve (12.3%) Evolut R (9.2%) Evolute Pro (1.0%) Lotus (0.7%)	Balloon-expandable (76.8%) self-expanding (22.5%) mechanically expandable (0.7%)	Transfemoral (96.4%)	30 days after operation
Kofler et al.	2021	Germany	965	81.0 ± 5.9	51.0%	2012 to 2019	SAPIEN 3 (41.3%) Evolut R (12.5%) Evolut Pro (11.6%) Acurate neo (11.2%) SAPIEN XT (10.6%) Acurate (3.5%) Portico (3.3%) CoreValve (2.2%) Lotus (2.2%) Centera (1.0%) SAPIEN 3 Ultra (0.3%) Lotus Edge (0.2%)	Balloon-expandable (52.2%) self-expanding (45.4%) mechanically expandable (2.4%)	NA	Discharge
Koh et al.	2015	Netherlands	56	79.7 ± 6.1	58.9%	NA	SAPIEN (100%)	Balloon-expandable (100%)	Transfemoral (NA) (majority)	3–5 days after procedure
Larroche et al.	2020	France	352	84.4 ± 6.7	54.3%	March 2011 to June 2016	CoreValve (40.9%) Evolut R (2.0%) SAPIEN 3 (28.1%) SAPIEN XT (29.0%)	Self-expanding (42.9%) balloon-expandable (57.1%)	Transfemoral (69.9%) transaortic (23.8%) transapical (30.1%) subclavian (3.4%)	Post-procedural
Mihara et al.	2015	United States	202	84.0 ± 8.4	48.0%	December 2010 to July 2012	SAPIEN (NA) SAPIEN XT (NA)	Balloon-expandable (100%)	Transfemoral (91.6%) transapical (7.9%) transaortic (0.5%)	Post-procedural
Musallam et al.	2021	United States	275	81.3 ± 8.2	50.6%	2013 to 2017	Evolut R (70.5%) SAPIEN S3 (29.5%)	Balloon-expandable (70.5%) self-expanding (29.5%)	Transfemoral (91.3%) transapical (NA) transaortic (NA)	Discharge
Park et al.	2018	South Korea	85	77.2 ± 7.1	50.6%	January 2011 to December 2015	NA	Self-expanding (100%)	Transfemoral (100%)	Post-procedural
Rys et al.	2018	Poland	40	79.9 ± 6.4	60.0%	NA	CoreValve (100%)	Self-expanding (100%)	Percutaneous (100%)	Post-procedural
Sakrana et al.	2016	Saudi Arabia	108	75.5 ± 11.8	27.8%	February 2013 to June 2015	SAPIEN XT (19.5%) SAPIEN 3 (8.3%) CoreValve (72.2%)	Balloon-expandable (27.8%) self-expanding (72.2%)	Transfemoral (94.4%) subclavian (5.6%)	Discharge
Seiffert et al.	2016	Germany	537	81.3 ± 6.2	52.3%	January 2012 to December 2013	SAPIEN XT (47.3%) CoreValve (22.9%) JenaValve (11.5%) Medtronic Engager (10.5%) Symetis Acurate (7.8%)	Balloon-expandable (47.3%) self-expanding (52.7%)	Transfemoral (53.8%) transapical (46.2%)	Discharge
Unbehaun et al.	2012	Germany	358	79.5 ± 8.3	66.5%	April 2008 to March 2011	SAPIEN (100%)	Balloon-expandable (100%)	Transapical (100%)	Post-procedural

PVL, paravalvular leakage; TAVR, transcatheter aortic valve replacement.

**Figure 2:**
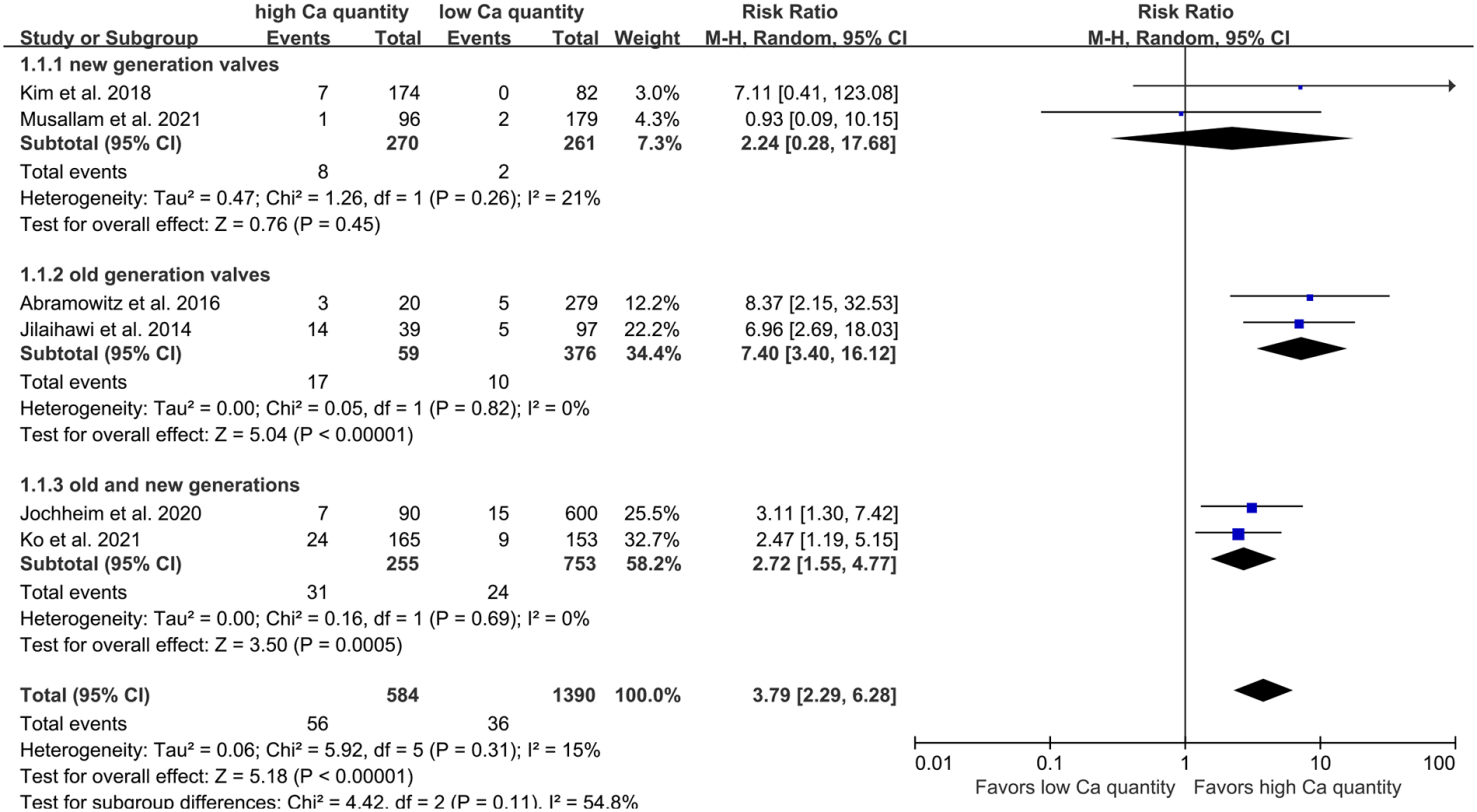
Risk of PVL in patients with aortic valve calcification after TAVR. RR, relative risk; PVL, paravalvular leakage; TAVR, transcatheter aortic valve replacement; M-H, Mantel-Haenszel.

**Figure 3:**
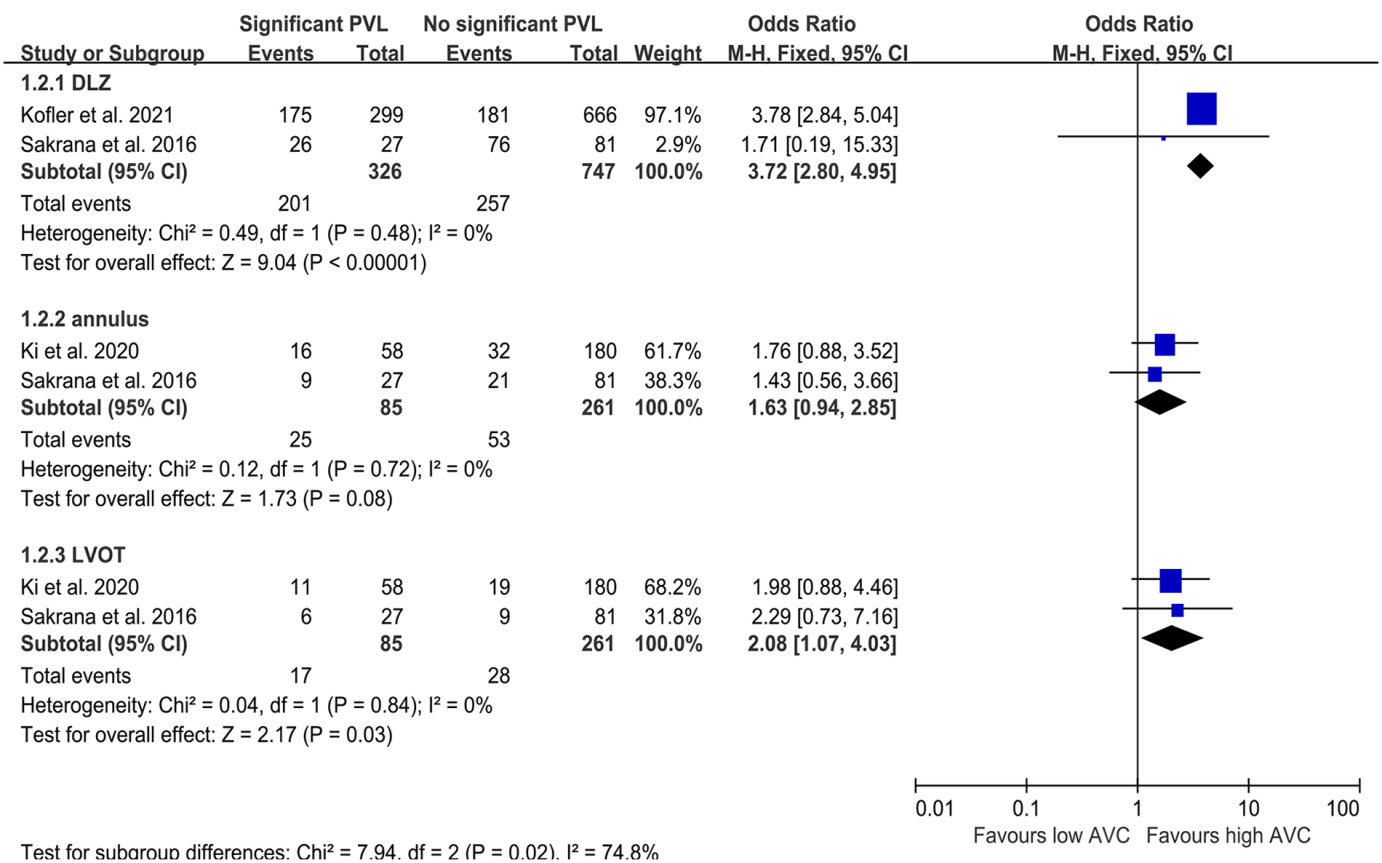
Odds ratio of PVL in patients with aortic valve calcification after TAVR. OR, odds ratio; PVL, paravalvular leakage; TAVR, transcatheter aortic valve replacement; M-H, Mantel-Haenszel; DLZ, device landing zone; LVOT, left ventricular outflow tract.

Six studies were included to evaluate the RR of significant PVL in patients with a high quantity of calcium. Kim et al. (20) defined the PVL in 1 year, while the duration was shorter in other studies. Overall, the percentage of high quantity of calcium in patients included in this analysis was 29.6%, and the risk of significant PVL ranged from 0% to 5.9% and 1.0% to 35.9% in patients without or with a high quantity of calcium, respectively. High quantity of calcification was observed to be significantly associated with PVL when valves without sealing skirt (RR 7.40, 95% CI 3.40–16.12; *P* < 0.001; Figure 2), while no such effect was observed for valves with sealing skirt (RR 2.24, 95% CI 0.28–17.68; *P* = 0.26; Figure 2). The total pooled results demonstrated a higher risk of significant PVL in patients with a high quantity of calcification compared to those with a low quantity (RR 3.79, 95% CI 2.29–6.28; *P* < 0.001; Figure 2). Heterogeneity across studies was observed (*I*^2^ = 15%). When the results from three studies were pooled, calcification in the DLZ or LVOT was found to have a significant effect on PVL (OR 3.72, 95% CI 2.80–4.95; *P* < 0.001; OR 2.08, 95% CI 1.07–4.03; *P* < 0.05; Figure 3, respectively), while no effect was observed for calcification in the annulus (OR 1.63, 95% CI 0.94–2.85; *P* = 0.08; Figure 3).

**Figure 4:**
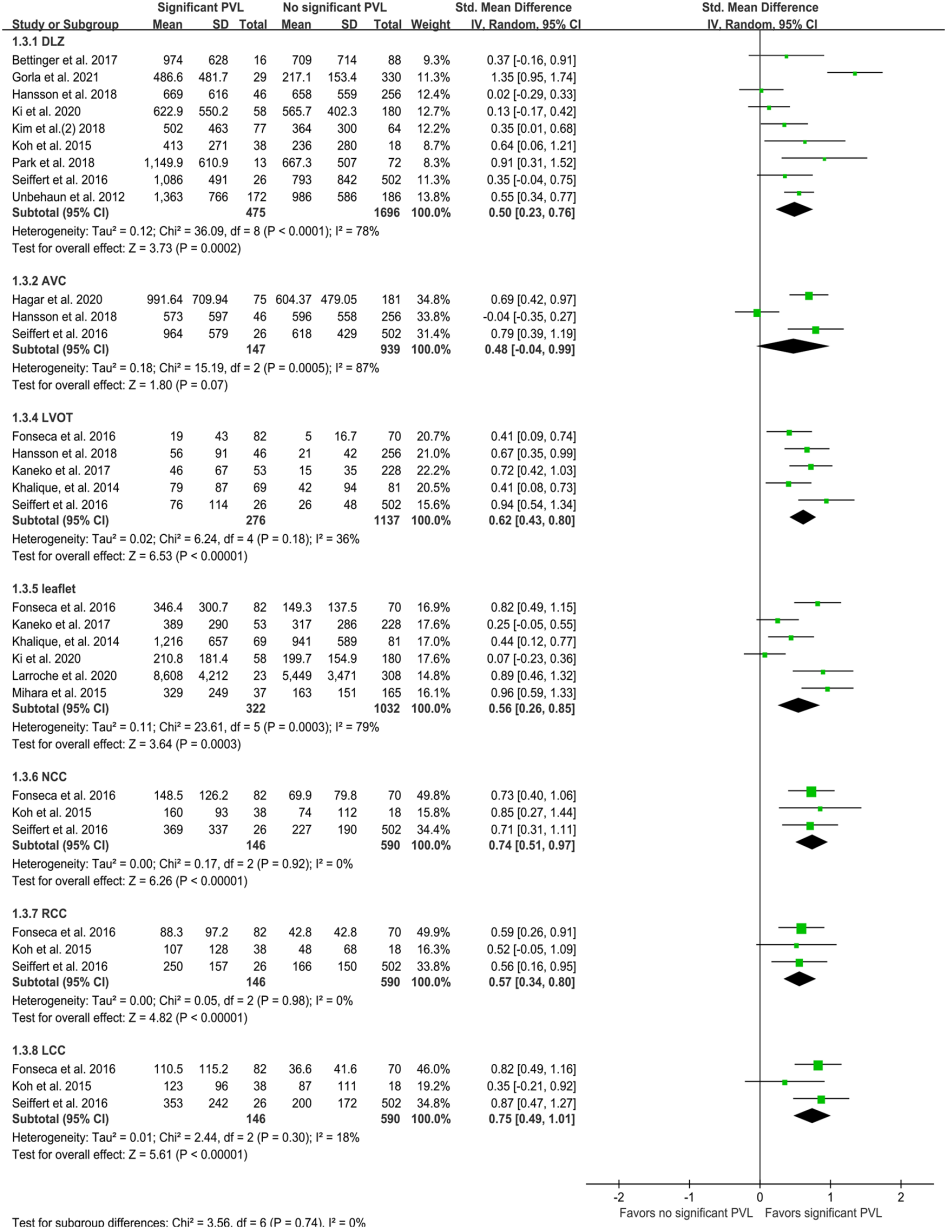
Quantity of calcification (mm^3^) per region. PVL, paravalvular leakage; IV, inverse variance; DLZ, device landing zone; AVC, aortic valve complex; LVOT, left ventricular outflow tract; NCC, non-coronary cusp; RCC, right coronary cusp; LCC, left coronary cusp.

**Figure 5:**
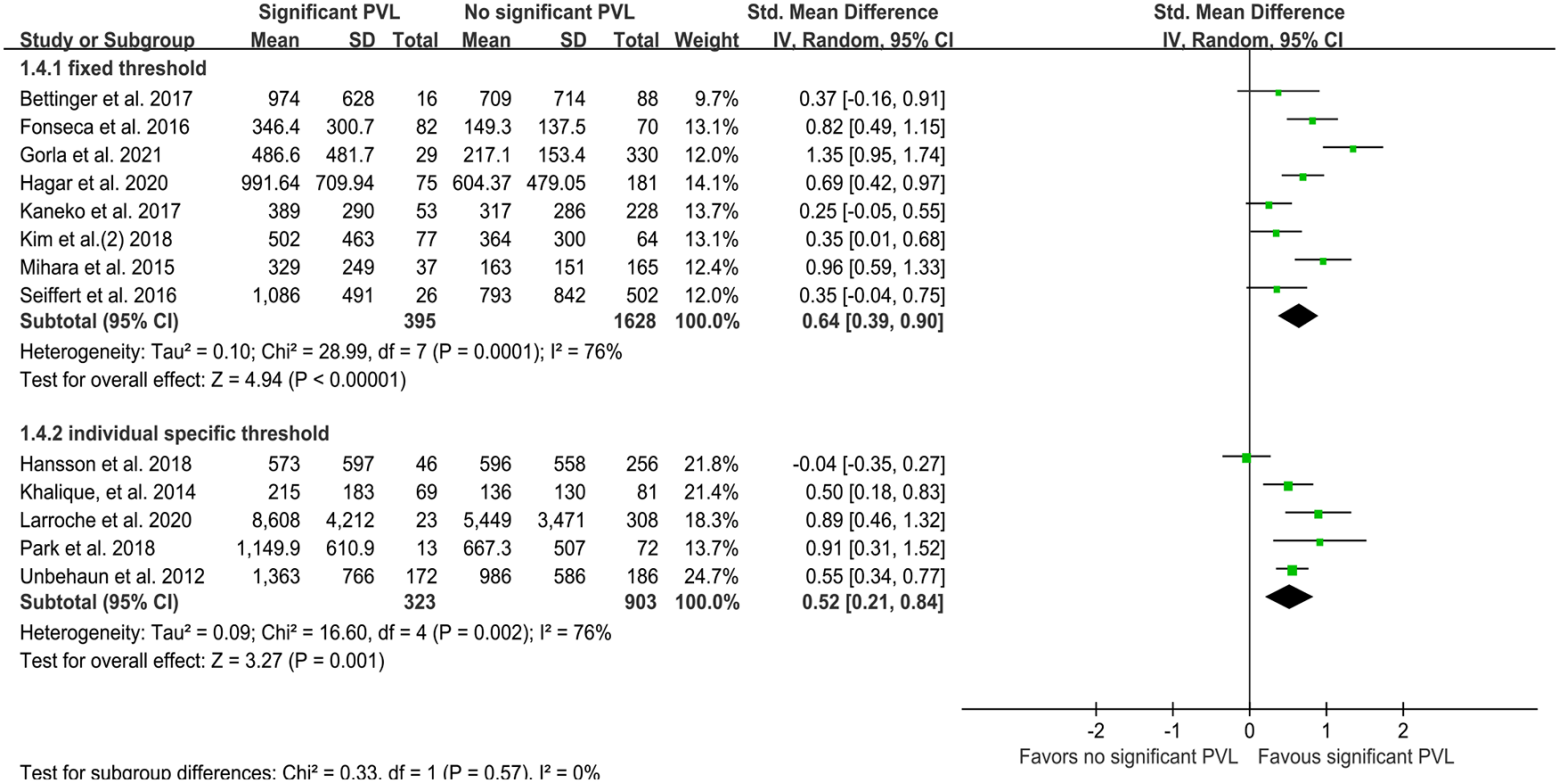
Quantity of calcification (mm^3^) per MDCT detected threshold type. MDCT, multidetector computed tomography; PVL, paravalvular leakage; IV, inverse variance.

**Figure 6:**
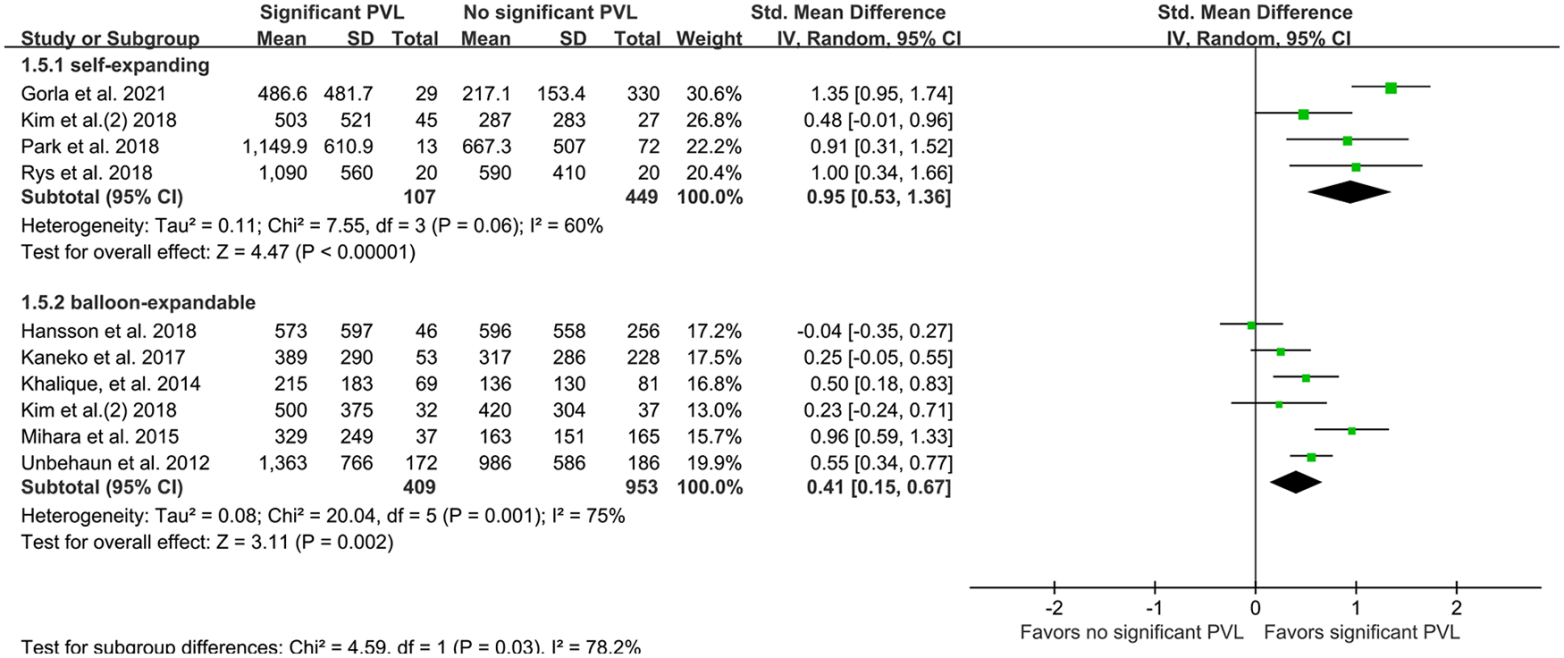
Quantity of calcification (mm^3^) per expandable type. PVL, paravalvular leakage; IV, inverse variance.

High quantities of calcification in the DLZ, LVOT, leaflets, and each cusp were associated with significant PVL (Figure 4), while no such effect was observed for calcification in the AVC (SMD 0.48, 95% CI −0.04–0.99; *P* = 0.07; Figure 4). On pooling the results of 13 studies, calcium quantity was found to be associated with PVL regardless of whether an individual-specific or fixed threshold was used for MDCT (Figure 5). Furthermore, the effect of aortic valve calcification on PVL after TAVR was similar for both self-expanding and balloon-expandable devices, both showing a significant effect on PVL (Figure 6).

The present analysis suggested that PVL after TAVR was associated with the quantity of aortic valve calcification, especially in the LVOT, valve leaflets, and DLZ. Moreover, a high quantity of calcium was associated with PVL, regardless of whether a self-expanding/balloon-expandable valve was used or if a fixed/patient-specific detection threshold was used.

The clinical application of TAVR is currently being expanded to the treatment of younger and lower-risk patients with aortic stenosis (21, 22); however, PVL after TAVR is associated with adverse outcomes (23). Aortic valve calcification may result in PVL; thus, it was important to explore their relationship in detail. We pooled the results of recent studies and performed subgroup analyses by new or old generation valve, location, expandable type, and MDCT threshold type. To our knowledge, this is the first meta-analysis to analyze the effect of the quantity and location of aortic valve calcification on PVL after TAVR.

The effect of calcification on PVL after TAVR has remained controversial. It has been reported that the volume of aortic calcification influences PVL (7, 8, 24, 25); however, some studies suggest that the degree of calcification does not influence the likelihood of PVL occurrence after TAVR (26, 27). The present meta-analysis explored the effect of the quantity and location of calcification on PVL using the following three analyses: RR, OR, and SMD. We found that the quantity of calcification in the LVOT increases the risk of PVL after TAVR. The OR and SMD analyses suggested that calcification located in the annulus does not have a significant effect on PVL. It appeared that prosthesis implantation eliminates the obstructive characteristics of annulus calcification, while calcium in the LVOT remains unaffected (28). It is possible that the perfect anchoring of the prosthesis implantation could have prevented the calcification at the aortic valve annulus.

When assessing calcification using MDCT, Kofler et al. reported the use of individual-specific Hounsfield unit (HU) thresholds instead of the arbitrarily defined HU thresholds (29). The choice of an empirical, fixed threshold does not consider the variation in the canal attenuation between patients (30). However, we observed high levels of calcification significantly associated with PVL even after examining the patients with a fixed threshold. This may be because of the high quantity of calcification and minimal error caused by the examination threshold. In addition, Fonseca et al. found that the volume of calcium observed using a threshold of 850 HU is the best predictor of PVL (31). Therefore, we believe that for patients with high levels of calcification, it may be more appropriate to fix the threshold as this simplifies image processing.

Recent studies have reported that PVL after TAVR is more common in patients with self-expanding prostheses than in those with balloon-expandable prostheses (32). In contrast, other studies have reported no significant association between prosthesis expandable type and the incidence of PVL (33). We observed that a high quantity of calcium is associated with PVL regardless of the use of self-expanding or balloon-expandable prostheses. Therefore, the use of such devices may not improve PVL to a great extent in patients with high calcification. For these patients, next-generation devices may be more effective as newer methods, such as anchoring or sealing, repositioning, and inflatable cuffs, are used (7, 34) with added leakage-proof function (35).

Plus, subgroup analysis was conducted with the presence or absence of sealing skirts. Abramowitz et al. (36) and Jilaihawi et al. (37) conducted their studies only using old generation valves, while Kim et al. (20) and Musallam et al. (26) only used valves with sealing skirts, other two studies by Jochheim et al. (28) and Ko et al. (38) used both valves with and without sealing skirts. We observed that high quantity of calcification was associated with PVL significantly when only old generation valves were applied or both valves with and without sealing skirts were used, while no such effect was observed for valves with sealing skirt were used (RR 2.24, 95% CI 0.28–17.68; *P* = 0.26; Figure 2). Hence, we infer that sealing skirt has a marvelous effect on reducing the incidence of PVL, even for patients with high calcific quantity. However, the valve with skirt probably has extremely high requirements for the operator to release the valve at proper position (39).

With the increasing research evidence on calcification and PVL, we have found that the description of calcification is becoming more refined, and even computer-based calcification modeling is now being used. Moreover, valve replacement techniques are more diverse and patient-specific. As such, a quantitative account of these studies is challenging, and that we would be providing a qualitative overview of the related evidence and advances in the field. On one hand, research on computer models is rapidly progressing. Specifically, fluid-structure interaction (FSI) modeling can accurately simulate the effect of aortic valve calcification on PVL (40–42); in bicuspid aortic valve replacement due to calcification, a finite element model suggested that aligning the bioprosthetic commissures with the native commissures yielded the lowest PVL (41, 43). On the other hand, the description of calcified plaque features and PVL features is becoming increasingly accurate; however, interestingly, the related conclusions seem to be inconsistent. The aortic valve calcium scores for NCC, RCC, and NCC/RCC showed a significant relation with PVL located in the cusps of the aortic valve. Meanwhile, the scores for RCC and RCC/LCC showed a significant relation with PVL located in the commissures (44). Reportedly, with the use of SAPIEN 3 Ultra balloon-expandable valves, the amount of calcification on the leaflets is not related to PVL; only the Eccentricity Index affects the incidence of PVL (45). This index is calculated as the maximum absolute difference in calcium volume between the leakage sectors for AVC/LVOT; it describes asymmetrical calcium load (45). In addition, the use of progressively more advanced prosthetic valves may lower the incidence of PVL in patients with calcification, but it may involve higher costs. According to reports by Piayda et al., patients who received Evolut R and Pro treatment, even with severe calcification, did not experience PVL recurrence (46). Nevertheless, a computer simulation showed that Evolut Pro reduced PVL by half compared with Evolut R (43). Ong et al. discovered that Edwards SAPIEN 3 Ultra provides excellent performance in patients with significant valvular calcification (47). Theoretically, a meta-analysis on more refined calcification characteristics to single valve type is warranted. However, given the lack of consensus between relevant clinical studies to date, relevant data are scarce and limiting in this sense.

Our present meta-analysis has several limitations. All selected studies were retrospective, most being single-center observational studies. Moreover, subgroup analysis was performed for a small number of studies, which may induce bias. In addition, owing to the lack of available data, we did not perform a pooled analysis of the effect of the more specific location of calcification on PVL according to a more detailed classification of the valves.

This meta-analysis provided further evidence that aortic valve calcification—including its quantity and location—can negatively impact PVL. Our findings support the clinical relevance of developing preventive measures for PVL, particularly in patients with severe aortic valve calcification. Nevertheless, further studies are needed to develop more optimized and uniform treatment strategies to prevent PVL. The need for these studies is urgent as TAVR may soon become an important tool for the treatment of aortic stenosis.

## 3. Conclusions

Altogether, our present study elucidated the effect of aortic valve calcification on PVL and showed that the quantity and location of aortic valve calcification can help predict PVL. Furthermore, our results provide a reference for the selection of MDCT thresholds before TAVR. We also showed that balloon-expandable valves may not be effective in patients with high calcification, and valves with sealing skirts instead of those without sealing skirts should be applied more to prevent PVL from happening.

## Data Availability

The original contributions presented in the study are included in the article/**Supplementary Material**, further inquiries can be directed to the corresponding author/s.
