# Supplementary material for: Quantity and location of aortic valve calcification predicts paravalvular leakage after transcatheter aortic valve replacement: a systematic review and meta-analysis
Source: Front Cardiovasc Med. 2023 May 24;10:1170979. doi: 10.3389/fcvm.2023.1170979 (PMC10244734; doi:10.3389/fcvm.2023.1170979)
Supplement: Supplementary file 1 [file Datasheet1.docx]

Supplementary Material

# Supplementary Figures


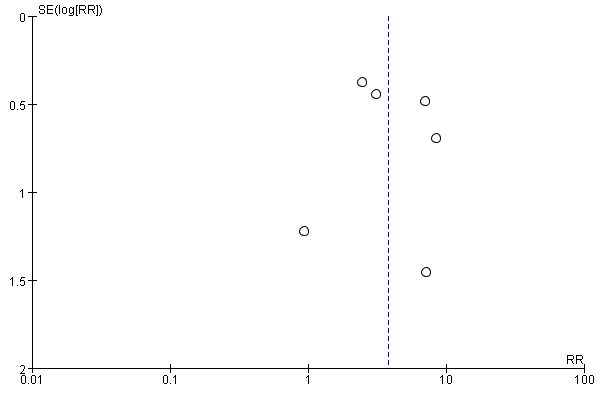


**Supplementary Figure 1.** Funnel plot of risk of PVL in patients with aortic valve calcification after TAVR.


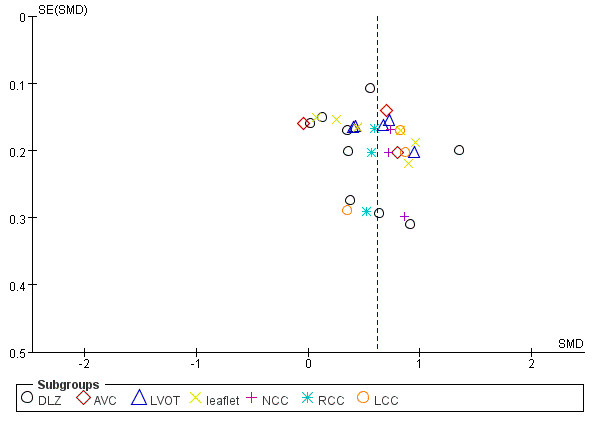


**Supplementary Figure 2.** Funnel plot of quantity of calcification per region.


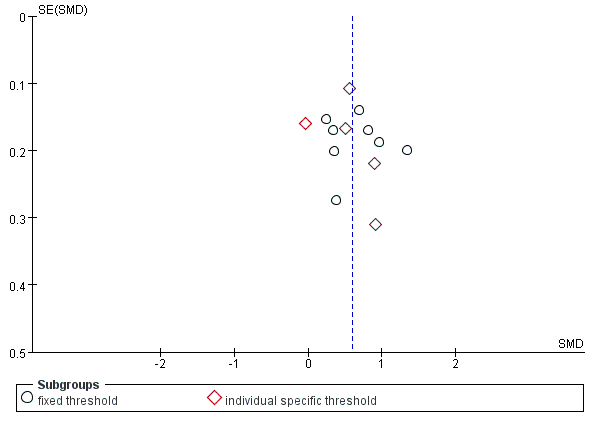


**Supplementary Figure 3.** Funnel plot of quantity of calcification per MDCT detected threshold type.


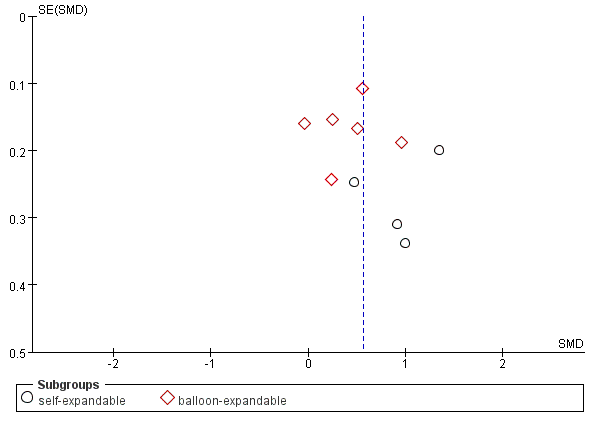


**Supplementary Figure 4.** Funnel plot of quantity of calcification per expandable type.
